# Favored subjects and psychosocial needs in music therapy in terminally ill cancer patients: a content analysis

**DOI:** 10.1186/s12904-016-0122-7

**Published:** 2016-05-12

**Authors:** Pia Preissler, Sarah Kordovan, Anneke Ullrich, Carsten Bokemeyer, Karin Oechsle

**Affiliations:** Department of Oncology, Hematology and BMT with section of Pneumology, University Medical Center Eppendorf, Hamburg, Germany; Department of Medical Psychology, University Medical Center Eppendorf, Hamburg, Germany

**Keywords:** Music therapy, Subjects, Needs, Psychosocial, Cancer, Terminally ill, Palliative care

## Abstract

**Background:**

Research has shown positive effects of music therapy on the physical and mental well-being of terminally ill patients. This study aimed to identify favored subjects and psychosocial needs of terminally ill cancer patients during music therapy and associated factors.

**Methods:**

Forty-one Patients receiving specialized inpatient palliative care prospectively performed a music therapy intervention consisting of at least two sessions (total number of sessions: 166; per patient average: 4, range, 2–10). Applied music therapy methods and content were not pre-determined. Therapeutic subjects and psychosocial needs addressed in music therapy sessions were identified from prospective semi-structured “field notes” using qualitative content analysis. Patient- and treatment-related characteristics as well as factors related to music and music therapy were assessed by questionnaire or retrieved from medical records.

**Results:**

Seven main categories of subjects were identified: “condition, treatment, further care”, “coping with palliative situation”, “emotions and feelings”, “music and music therapy”, “biography”, “social environment”, and “death, dying, and spiritual topics”. Patients addressed an average of 4.7 different subjects (range, 1–7). Some subjects were associated with gender (*p* = .022) and prior impact of music in patients’ life (*p* = .012).

The number of subjects per session was lower when receptive music therapy methods were used (*p* = .040). Psychosocial needs were categorized into nine main dimensions: “relaxing and finding comfort”, “communication and dialogue”, “coping and activation of internal resources”, “activity and vitality”, “finding expression”, “sense of self and reflection”, “finding emotional response”, “defocusing and diversion”, and “structure and hold”. Patients expressed an average of 4.9 psychosocial needs (range, 1–8). Needs were associated with age, parallel art therapy (*p* = .010), role of music in patient’s life (*p* = .021), and the applied music therapy method (*p* = .012).

**Conclusion:**

Seven main categories of therapeutically relevant subjects and nine dimensions of psychosocial needs could be identified when music therapy was delivered to terminally ill cancer patients. Results showed that patients with complex psychosocial situations addressed an average number of five subjects and needs, respectively. Some socio-demographic factors, the role of music in patient’s lives and the applied music therapy methods may be related with the kind and number of expressed subjects and needs.

## Background

Palliative care comprises comprehensive care for patients with incurable advanced diseases and their relatives respecting physical, psychological, social and spiritual needs. Therefore, palliative care includes medical, psychosocial and spiritual interventions that are carried out by a multi-professional team. In the last years, music therapy has been increasingly integrated into multi-professional palliative care. Case series, qualitative analyses, retrospective and single-arm interventional studies have suggested beneficial effects of music and music therapy on pain, dyspnea, physical comfort, body perception, anxiety, and mood [1–9]. In addition, patients reported more communication about spiritual issues and feel more that their spiritual needs are respected adequately when receiving music therapy during palliative care [7]. Randomized studies demonstrated improved quality of life, pain reduction as well as less anxiety, tiredness and drowsiness [10–12], but review analyses found profound data only for effects on quality of life and pain perception in patients receiving end-of-life care [13–15].

There is only very limited systematic information on feasibility and efficacy of different music therapy methods and recommendations for their adequate application in terminally ill patients [16]. In particular, systematic data on issues, topics, and needs that can or should be included into music therapy seriously in terminally ill patients are not sufficiently investigated until today. This is of particular importance as other data indicates potential critical effects of music therapy in palliative care, e.g., increasing the patients` vulnerability, caused by insensitive or inadequate delivery [17]. Therefore, systematic knowledge about topics and needs of high relevance for patients which have to be addressed sensitively are crucial to develop effective music therapy interventions in palliative and end-of-life care.

The aims of this study was to address this gap and (i) to explore subjects and psychosocial needs expressed by terminally ill cancer patients in specialized inpatient palliative care during music therapy and (ii) to assess factors associated with the number and kind of these subjects and needs.

## Methods

### Study design

Data were collected in a prospective interventional study that was conducted in inpatient palliative care setting. The primary aim of the analysis presented here was to explore and quantify the various therapeutic subjects and psychosocial needs that terminally ill cancer patients bring up during music therapy. Secondary study aims were to identify the impact of socio-demographic and treatment-related patient characteristics, previous experience with music and music therapy and the use of further psychosocial support during inpatient palliative care.

### Study population

The study included a convenience sample of advanced cancer patients admitted to the specialized inpatient palliative care unit (henceforth referred to as SPCU) of the University Medical Center Hamburg-Eppendorf, Germany. Presence of significant physical and/or psychosocial symptoms prohibiting further care at home or in non-specialized inpatient wards were criteria for referral to SPCU.

Patients were included if they met the following criteria:

- advanced cancer
- palliative care in SPCU between June 2012 and October 2014
- older than 18 years
- given written informed consent for study participation, data analysis and publication.

Exclusion criteria were:

- inadequate knowledge of German language
- insufficient cognitive function.

Eligible patients were recruited within 48 h after admission to SPCU. The study protocol was approved by the local ethics committee of the General Medical Council of Hamburg (PV4053, 10 April 2012).

### Music therapy intervention

The intervention was carried out by the first authors who both work as trained music therapists in the SPCU’s multi-professional team. Music therapy was carried out in individual sessions (one-on-one therapy) using a variety of music therapy techniques that can be categorized as receptive and active methods. For study eligibility, music therapy intervention had to consist of at least two sessions. The first session was offered to the patient within two working days after admission to the SPCU. The intervention ended with the patient`s discharge, his/her death or withdrawal of consent. Patients could determine frequency (1–3 sessions per week) and duration (20–90 min) of therapy sessions, and could make choices on music therapy techniques and contents in each session (see Table 1). Details on the intervention and the evaluation of feasibility, favored techniques, beneficial effects, and factors associated with these outcomes are presented elsewhere [18].

**Table 1:** Music therapy intervention and techniques used within the intervention

Music therapy intervention		
● Starting within 48 h after admission to the specialized inpatient palliative care unit (SPCU) ● One-on-one music therapy carried out by trained music therapists ● Extent of music therapy (within given limits), applied techniques and content of sessions determined by patients		
Applied music therapy methods and respective techniques		
● Receptive, active, combined (active/receptive), therapeutic conversation only		
Receptive	Active	Therapeutic conversation only
● Listening to relaxing music (performed by the therapist) ● Listening to music (performed by the therapist) related to therapeutic issues ● Music-led imagination or Guided Imagery and Music	● Instrumental improvisation (patient individually or together with therapist) ● Singing songs	● Verbal discussion of instruments, music’s role in patient’s life/musical biography and other music-related themes ● Verbal expression and processing of thoughts and feelings

### Data collection methods

Subjects and psychosocial needs addressed by the patients during music therapy were documented in “field notes”, which are detailed accounts of the sessions’ content. Therefore, notes were immediately taken after each session by the treating music therapists. Field notes were semi-structured meaning that the involved therapists answered the following key questions for each patient and session: duration, music therapy techniques applied, therapeutic subjects and psychosocial needs addressed by the patient, and particularities.

In order to obtain socio-demographic data, information on the previous impact of music on patients’ life, and previous experience with music therapy, patients answered a self-report questionnaire at the beginning of the music therapy intervention. Medical data and additional data on psychosocial support received during inpatient palliative care were taken from medical records.

### Statistical analysis

To gain in-depth understanding of therapeutic subjects and psychosocial needs of patients, all field note transcripts were analyzed by the two first authors using qualitative content analysis. Content analysis comprises a systematic coding and categorization of narrative materials in order to identify patterns [19, 20]. This approach allows to analyze data qualitatively and also to interpret quantitative counts of the codes [21]. Therefore, based on the transcripts, the two first authors developed a preliminary coding frame using inductive coding strategies, and discussed it with each other. With subsequent transcripts, codes were added and revised until no new key themes emerged [22]. Therapeutic subjects and psychosocial needs were categorized independently by the two first authors using the final coding frame. In cases of differences, the respective data were discussed by the two first authors until consensus was found.

With respect to quantitative statistics, descriptive analyses were conducted to examine sample characteristics, and the distribution of subjects and needs in the course of music therapy. To gain knowledge about possible changes in frequencies of subjects and needs (comparing first vs. fourth session), Chi^2^-tests were calculated. Spearman’s tests were used to examine bivariate associations of subjects or needs with patient characteristics and music-related factors. All significance tests were two-tailed using a significance level of α < .05. Quantitative analyses were performed using the statistical package SPSS Statistics software version 22.0 (IBM, USA).

## Results

### Patient characteristics and music therapy intervention

A total of 41 terminally ill cancer patients admitted to the SPCU took part in this study, ranging in age from 36 to 89 years (median age of 64 years). The patients stayed for a median of 12 days on the ward (standard deviation (SD) 13.1; range, 5–27 days). Six patients (12 %) died later on the ward while 35 patients (88 %) were discharged home or to hospice care. Detailed patient-related factors are presented in Table 2.

**Table 2:** Patient-related factors

	Patients (*N* = 41)	
	n	%
Age: median/average/SD/range	64/63.2/12.3/36–89	
Gender		
Male	10	24
Female	31	76
Tumor diagnosis		
Lung cancer	9	22
Gastrointestinal tumors	14	34
Urologic tumors	6	15
Gynecologic tumors	10	24
Other	2	5
Partnership		
Yes	26	63
No	15	37
Children		
None	16	39
One child	7	17
Two children	15	36
Three or rmore	3	7
Living situation		
Living alone	16	39
Living in a family	23	56
Living in a nursery home	2	5
Religion		
No religion	13	32
Religious	8	20
Did not want to answer	20	49
Additional psychosocial care		
Social care	41	100
Psycho-oncology	14	34
Art therapy	22	54
Previous experience with music therapy	4	10
“Music is relevant in my life”	21	52
Playing an instrument (previously or ongoing)	19	47
Singing regularly	18	46

*Abbreviations*: *SD* standard deviation

A total number of 166 music therapy sessions were performed in these 41 patients with an average of four sessions per patient (SD 1.9; range, 2–10). Average duration per session was 41 min (SD 11.4; range, 20–70). Receptive music therapy was applied in 45 % of sessions (74/166), active music therapy in 25 % (41/166), a combination of both in 7 % (12/166), and therapeutic conversation only in 23 % (38/166).

### Therapeutically relevant subjects addressed during music therapy intervention

In total, 469 therapeutically relevant subjects were brought up during all 166 music therapy sessions resulting in an average number of 2.8 per session and patient (range, 1.0–3.3).

Qualitative content analysis revealed seven main categories of therapeutically relevant subjects: “condition, treatment, further care” in 21 % of all mentioned subjects (100/469), “coping with palliative situation” in 21 % (99/469), “emotions and feelings” in 19 % (90/469), “music and music therapy” in 15 % (70/469), “biography” in 9 % (43/469), “social environment” in 9 % (41/469), and “death, dying, and spiritual topics” in 6 % (26/469).

Frequencies of subject categories were varying during the course of music therapy sessions, but did not show any statistically significant trend. Table 3 presents the course of the seven subject categories during the first four music therapy sessions since this number of sessions was applied in about half of all patients.

**Table 3:** Course of therapeutic subjects during the first four music therapy sessions

	Session 1^a^	Session 2^a^	Session 3^b^	Session 4^c^	Session 1 vs. Session 4
	145 subjects (%)	91 subjects (%)	72 subjects (%)	44 subjects (%)	*p*-value^d^
Condition, treatment, further care	15	19	26	20	.527
Coping with palliative situation	17	24	26	30	.075
Emotions and feelings	18	24	17	14	.617
Music and music therapy	19	10	11	9	.113
Biography	11	10	11	11	.843
Social environment	12	5	4	14	.734
Death, dying, spiritual topics	8	8	4	2	.173

*Abbreviations*: *pts* patients

^a^ *n* = 41 pts, ^b^ *n* = 34 pts, ^c^ *n* = 20 pts; decrease of sample size due to variability of frequency of music therapy sessions. Application of two sessions was minimum requirement for study eligibility; ^d^Chi^2^-test

The 41 patients brought up an average number of 4.7 subjects of the seven main categories (range, 1–7) during their total music therapy intervention. Subjects of the category “condition, treatment, further care” were prevalent in 85 % of patients (35/41) at least in one music therapy session, “coping with palliative situation” in 80 % (33/41), “emotions and feelings” in 68 % (28/41), “music and music therapy” in 78 % (32/41), “biography” in 61 % (25/41), “social environment” in 63 % (26/41), and “death, dying, and spiritual topics” in 37 % (15/41).

The seven main categories of subjects contained 34 sub-categories which are presented in detail in Table 4.

**Table 4:** Course of main subject categories and sub-categories during the first four sessions

	Session 1 (*n* = 41 pts)	Session 2 (*n* = 41 pts)	Session 3 (*n* = 34 pts)	Session 4 (*n* = 20 pts)
Condition/treatment/further care	22 (54 %)	17 (41 %)	19 (56 %)	8 (40 %)
Current condition	11 (50 %)	8 (47 %)	9 (47 %)	4 (44 %)
Course of disease and treatment	7 (32 %)	6 (35 %)	5 (26 %)	2 (23 %)
Further care following SPCU	4 (18 %)	3 (18 %)	5 (26 %)	2 (23 %)
Coping with palliative situation	24 (59 %)	23 (56 %)	19 (56 %)	13 (65 %)
Self-reflection	6 (24 %)	7 (30 %)	7 (37 %)	2 (15 %)
Personal resources	5 (20 %)	5 (22 %)	6 (32 %)	1 (8 %)
Aims/plans/wishes for things to come	7 (28 %)	4 (17 %)	1 (5 %)	2 (15 %)
Coping strategies	4 (16 %)	3 (13 %)	2 (11 %)	3 (23 %)
Recognition and dealing with needs	2 (8 %)	–	1 (5 %)	2 (15 %)
Personal and other’s boundaries	–	2 (9 %)	2 (11 %)	3 (23 %)
Life review	1 (4 %)	2 (9 %)	–	–
Emotions and feelings	26 (63 %)	22 (54 %)	12 (35 %)	6 (30 %)
Grief and loss	8 (31 %)	6 (27 %)	4 (33 %)	4 (67 %)
Anger and distress	1 (4 %)	4 (18 %)	3 (25 %)	–
Anxiety	4 (15 %)	2 (9 %)	3 (25 %)	–
Hope	7 (27 %)	2 (9 %)	–	1 (17 %)
Ambivalent feelings	3 (12 %)	2 (9 %)	–	–
Feeling overwhelmed	1 (4 %)	2 (9 %)	–	–
Isolation	1 (4 %)	1 (4 %)	2 (27 %)	–
Feeling of insecurity	1 (4 %)	2 (9 %)	–	–
Sense of confidence	–	1 (4 %)	–	1 (17 %)
Music and music therapy	27 (66 %)	10 (24 %)	8 (24 %)	9 (45 %)
Musical anamnesis/history	20 (74 %)	5 (55 %)	2 (25 %)	5 (56 %)
Experiences with music therapy	3 (10 %)	1 (11 %)	3 (38 %)	3 (33 %)
Aims/wishes related to music therapy	2 (7 %)	3 (33 %)	3 (38 %)	1 (11 %)
Questions about music therapy techniques and instruments	2 (7 %)	1 (11 %)	–	–
Biography	16 (39 %)	9 (22 %)	8 (24 %)	5 (25 %)
Profession and hobbies	9 (56 %)	4 (44 %)	2 (25 %)	2 (40 %)
Positive life events	2 (13 %)	3 (33 %)	2 (25 %)	2 (40 %)
Distressing life events	3 (18 %)	2 (22 %)	2 (25 %)	–
Family issues	2 (13 %)	–	1 (13 %)	–
Childhood experiences	–	–	1 (13 %)	1 (20 %)
Social environment	18 (44 %)	5 (12 %)	3 (8 %)	6 (30 %)
Family	10 (56 %)	2 (40 %)	2 (67 %)	2 (33 %)
Partnership, marriage	–	1 (20 %)	1 (33 %)	2 (33 %)
Social context and integration	8 (44 %)	2 (40 %)	–	2 (33 %)
Death/dying/spiritual topics	11 (27 %)	7 (17 %)	3 (8 %)	1 (5 %)
Death and dying	5 (45 %)	3 (43 %)	–	–
Spiritual and existential aspects	6 (55 %)	2 (29 %)	3 (100 %)	–
Farewell	–	2 (29 %)	–	1 (100 %)

*Abbreviations*: *pts* patients

### Psychosocial needs expressed during music therapy intervention

During all 166 music therapy sessions, patients expressed 417 psychosocial needs resulting in an average number of 2.5 needs per session and patient (range, 0.2–3.8). The various needs were categorized into nine main dimensions of psychosocial needs: “relaxing and finding comfort” in 19 % of all mentioned needs (79/417), “communication and dialogue” in 14 % (58/417), “coping and activation of internal resources” in 12 % (52/417), “activity and vitality” in 11 % (47/417), “finding expression” in 11 % (45/417), “sense of self and reflection” in 9 % (38/417), “finding emotional response” in 8 % (35/417), “defocusing and diversion” in 8 % (34/417), and “structure and hold” in 7 % (29/417).

The frequencies of the needs dimensions changed during the course of music therapy sessions, but only “coping and activation of internal resources” increased significantly from session one to four (*p* = .020). Detailed course of the needs dimensions during the first four music therapy sessions are presented in Table 5.

**Table 5:** Psychosocial needs during the first four music therapy sessions

	Session 1^a^	Session 2^a^	Session 3^b^	Session 4^c^	Session 1 vs. Session 4
	62 needs (%)	51 needs (%)	46 needs (%)	25 needs (%)	*p*-value^d^
Relaxing and finding comfort	12	17	23	12	.925
Communication and dialogue	23	12	13	12	.201
Coping and activation of internal resources	7	14	9	24	**.020**
Activity and vitality	11	10	9	12	.925
Finding expression	7	14	19	8	.796
Sense of self and reflection	11	11	6	4	.287
Finding emotional response	9	9	3	4	.378
Defocusing and diversion	12	5	10	16	.550
Structure and hold	7	8	8	8	.796

^a^ *n* = 41 pts, ^b^ *n* = 34 pts, ^c^ *n* = 20 pts; decrease of sample size due to variability of frequency of music therapy sessions. Application of two sessions was minimum requirement for study eligibility; ^d^Chi^2^-test

bold data = statistically significant

The 41 patients brought up an average number of 4.9 of the nine main dimensions of needs (range, 1–8) during their total music therapy intervention. Needs of the dimension “Relaxing and finding comfort” were verbalized at least once by 66 % of patients (27/41), “communication and dialogue” by 66 % (27/41), “coping and activation of internal resources” by 59 % (24/41), “activity and vitality” by 49 % (20/41), “finding expression” by 54 % (22/41), “sense of self and reflection” by 46 % (19/41), “finding emotional response” by 42 % (17/41), “defocusing and diversion” by 54 % (22/41), and “structure and hold” by 44 % (18/41).

### Factors associated with therapeutically relevant subjects and psychosocial needs

Correlation analyses showed that with an increasing number of music therapy sessions, the number of psychosocial needs during the total intervention increased significantly (*r* = .620; *p* < .001), while the number of subjects did not (*r* = .288; *p* = .071). The number of subjects addressed per session even decreased with an increasing number of sessions (*r* = .480; *p* = .001), while the number of needs per session was unchanged.

Concerning therapeutically relevant subjects, subjects of the category “social environment” were addressed significantly more often by male than female patients (*r* = .360; *p* = .022), but none of the other subjects was associated with any patient characteristics. Patients reporting that music played a relevant role in their life less often brought up biographic subjects (*r* = −.414; *p* = .012) and addressed a lower number of subjects per session (*r* = −.395; *p* = .017), while prior experience with music therapy was not related to the kind and number of subjects. In addition, the kind of subjects were not associated with music therapy methods (receptive/active). In contrast, the number of subjects per session was lower in sessions in which receptive methods were performed (*r* = −.326; *p* = .040). For additional psycho-oncological or art therapy support no significant correlations with the kind or number of subjects were observed.

Regarding psychosocial needs, younger patients showed a higher number of needs per session (*r* = −.411; *p* = .008). The number of needs was lower in patients for whom music plays an important role in their life (*r* = .493; *p* = .002). Needs of the dimension “communication and dialogue” were significantly more often reported by patients additionally undergoing art therapy (*r* = .400; *p* = .010), but significantly less frequent in patients stating that music plays an important role in their life (*r* = −.378; *p* = .021). Patients receiving receptive music therapy significantly more often expressed need for “relaxing and finding comfort” (*r* = .387; *p* = .012), while patients performing active methods expressed the need for “activity and vitality” (*r* = .370; *p* = .017). Patients asking for “defocusing and diversion” received more receptive therapy forms (*r* = .346; *p* = .027).

## Discussion

This study qualitatively explored therapeutically relevant subjects and psychosocial needs of 41 terminally ill cancer patients during a total of 166 music therapy sessions. In addition, associated socio-demographic and treatment-related characteristics, previous experience with music and music therapy, and the use of other psychosocial support were investigated. The analyses were carried out within a prospective interventional study on music therapy in SPCU.

Qualitative content analysis performed independently by two music therapists identified seven main categories of therapeutic subjects brought up by the patients: “condition, treatment, further care”, “coping with palliative situation”, “emotions and feelings”, “music and music therapy”, “biography”, “social environment”, and “death, dying, and spiritual topics”. These categories covered 21 to 6 % of all 469 subjects occurring during all sessions. The prevalence was clearly higher (85 to 37 %) when considering which subjects were addressed by the patients at least once during music therapy: “condition, treatment, further care” and “coping with palliative care” were most common, whereas “death, dying, and spiritual topics” was least prevalent. This difference can be explained by the high number of different subjects the patients brought up with an average number of 4.7 subjects during the total music therapy intervention and 2.8 subjects per session. These findings underline the complex situation of palliative care patients who have to deal with various issues when facing the last period of their life.

The number of subjects addressed was lower in sessions comprising receptive music therapy than in those using active methods. Receptive music therapy is known to help defocus and relax, find comfort or reduce tension [16]. In contrast, active methods might facilitate the non-verbal expression of thoughts and feelings, the expression of suppressed emotions, and open verbalization of even difficult issues.

The number of addressed subjects was lower in patients stating that music plays a relevant role in their life. It can be assumed that these patients focus more on the experience of music and self-reflection than on the articulation of various problems. These patients also addressed biographic subjects less frequently.

Neither prior experience with music therapy nor additional psycho-oncological or art therapy support were associated with the number or kinds of subjects during music therapy. Therefore, music therapy can be offered to all patients in SPCU despite of additional supportive therapies or prior music therapy experiences.

The only socio-demographic factor associated with subjects addressed by patients was “social environment” which was significantly more frequent in male than female patients.

Nine main dimensions of psychosocial needs were identified: “relaxing and finding comfort”, “communication and dialogue”, “coping and activation of internal resources”, “activity and vitality”, “finding expression”, “sense of self and reflection”, “finding emotional response”, “defocusing and diversion”, and “structure and hold” representing in decreasing order 19 to 7 % of all needs occurring during all sessions. A previous qualitative interview pilot study conducted in end-of-life care retrospectively asked patients to describe their subjective experience with receptive music therapy and most frequently stated experiences were “relaxing and calming”, “sensation that the body feels lighter”, and “generation of relaxing images and visualization” [2]. Respecting the limits of comparing prospective with retrospective data, “relaxing and finding comfort” seem to be a frequent need of patients during music therapy within palliative care.

In our study, needs of the dimension “relaxing and finding comfort” and “communication and dialogue” were most frequent and “finding emotional response” was least prevalent. Patients showed an average number of 4.9 psychosocial needs during the total intervention and 2.5 needs per session. Comparable to therapeutic subjects this corresponds to the high psychosocial burden that terminally ill patients face.

While the number of subjects addressed per session decreased with an increasing number of sessions, the number of psychosocial needs expressed per session did not change. This results in an increasing number of different needs expressed during the course of intervention. This emphasizes the complex situations of problems and needs palliative care patients have to cope with and indicates that psychosocial needs might change in the course of disease. However, the number of needs did not increase, strengthening the assumption that music therapy and psychosocial support have beneficial impact on terminally ill patients.

Needs of the dimension “communication and dialogue” were more often prevalent in patients who additionally received art therapy, but overall additional psychosocial support seems not to have a significant impact on subjects and needs during music therapy. Therefore, both therapeutic approaches may be performed parallel in terminally ill patients receiving multi-professional care.

Younger patients expressed a significantly higher number of psychosocial needs per session and asked more often for “defocusing and diversion”. The number and kind of psychosocial needs was not correlated with gender, but a significant association with the number of addressed subjects was observed. Overall, these findings demonstrate that socio-demographic factors have no systematic influence on subjects or needs during music therapy.

Some psychosocial needs were associated with the applied music therapy methods (receptive: “relaxing and finding comfort” and “defocusing and diversion”/active: “activity and vitality” and “finding expression”). This was not surprising as the applied methods were based on patients’ choices, and therefore it could be expected that methods reflected his/her needs. However, it shows that the use of certain methods is not primarily associated with subjects but with needs and it demonstrates the high adaptability of music therapy interventions to the patients’ needs situation.

This study comprises several strengths and weaknesses. In this study the impact of statistical analyses is limited by its explorative character. In addition, this study did not evaluate physical and psychological symptoms which were focused in previous studies [3–17], which significantly limits comparability. Concurrently, qualitative studies are rare [2] and this study is the first to qualitatively analyze therapeutic subjects and needs in the context of music therapy in SPCU. Therefore, it brings up new aspects on music therapy in palliative care which might represent the basis for further clinical studies and help to decide which therapeutic subjects and psychosocial needs should be considered in study protocols. With respect to qualitative research the study consists of a sufficient sample size (41 patients and 166 music therapy sessions), although the sampling technique (convenience sample) may limit the generalizability of the findings.

## Conclusion

In conclusion, seven main categories of therapeutically relevant subjects and nine dimensions of psychosocial needs could be identified in music therapy in terminally ill cancer patients in SPCU. Patients with a complex psychosocial situation addressed an average number of 4.7 different subjects and 4.9 needs during the intervention. Age, gender, the role of music in the patient`s life, and the applied music therapy methods are associated with specific needs and subjects addressed by these patients. Music therapists should be aware of a high psychosocial burden in palliative care patients resulting in a broad range of subjects and needs which need to be addressed.

### Ethics approval and consent to participate

The study protocol was approved by the local ethics committee of the General Medical Council of Hamburg (PV4053, 10 April 2012). All study participants gave written informed consent.

### Consent for publication

Not applicable.

### Availability of data and materials

Ethical restrictions and local data protection regulations do not allow publication of raw data. All relevant data for the conclusions are presented in the manuscript.

## Abbreviations

pts: patients
SPCU: specialized inpatient palliative care unit
